# Graphene absorber on an SOI chip for active and passive mode locking of lasers

**DOI:** 10.1038/s41598-025-93051-z

**Published:** 2025-03-19

**Authors:** Tom Reep, Cheng-Han Wu, Didit Yudistira, Steven Brems, Inge Asselberghs, Marianna Pantouvaki, Joris Van Campenhout, Dries Van Thourhout, Bart Kuyken

**Affiliations:** 1 Photonics Research Group, Department of Information Technology, Ghent University-imec, Technologiepark-Zwijnaarde 15, 9052 Gent, Belgium; 2 Imec, Kapeldreef 75, 3001 Leuven, Belgium

**Keywords:** Semiconductor lasers, Mode-locked lasers, Fibre lasers, Optical properties and devices

## Abstract

We demonstrate both passive and active mode-locking of fiber lasers using a graphene-based absorber integrated on a silicon photonics platform manufactured using a wafer-scale CMOS process. Passive mode-locking is achieved at a 28 MHz repetition rate, generating 1.7 ps optical pulses, while active mode-locking is demonstrated at repetition rates of 4 GHz and 10 GHz. This work demonstrates the potential of scalable graphene-based saturable absorbers for fiber laser locking and paves the way for future fully integrated mode-locked laser systems.

## Introduction

Mode-locked lasers produce optical pulse-trains, which in the frequency domain consists of equidistantly spaced laser lines locked in phase. These devices can be used for a variety of applications spanning telecommunications, gas spectroscopy, and biomedical fields^1–3^. These optical pulse trains can be generated in laser cavities through saturable absorption (passive mode-locking) or loss/gain modulation (active mode-locking) at integer multiples of the round-trip time in the cavity^4^.

Passively mode-locked lasers typically employ III/V semiconductor-based saturable absorbers (SA). In fiber based mode-locked lasers semiconductor saturable absorber mirror assemblies (SESAMs) are common^5^, while for on-chip (integrated) mode-locked lasers reverse-biased electrically isolated III/V semiconductor amplifier sections are commonly employed^6–8^. These SA assemblies are limited in optical bandwidth and have slow recovery times (around 2.5 ps), which can restrict mode-locked laser performance^8,9^.

Recent developments on integrated Titanium:sapphire^10^ and ion-implanted SiN and AlOx amplifying waveguides have demonstrated high on-chip gain (> 30 dB) and large saturation powers (> 21 dBm)^11,12^. While Q-switched lasers have been achieved with these amplifiers using Kerr-based saturable absorbers in SiN^13^, passive mode-locking of lasers has remained an ongoing challenge in part due to the lack of on-chip saturable absorbers.

Saturable absorbers based on 2D materials offer a promising solution to address this gap^14^. In particular, graphene, MoS_2_, and WS_2_ have demonstrated stable mode-locking performance^15–18^. Among these, graphene stands out due to its broadband operation, low saturation intensity, ultrafast recovery time (~200 fs), and tunable modulation depth^15,19^. Graphene-based passively mode-locked lasers have been realized using single- or few-layer flakes deposited on fiber ferrules^15,16^. However, these approaches face limitations in modulation depth due to the atomic thinness of 2D materials and limited control over material quality.

To address the issue of interaction length, graphene sheets have been placed in the evanescent tail of propagating modes in D-shaped and micro-fibers^20–22^. Additionally, low-speed (<300 Hz) electrical tuning of graphene’s nonlinear optical response has been demonstrated using ionic gels^23^. However, fiber-based devices lack scalability and are unsuitable for active mode-locking in fiber cavities due to the slow electro-optic response of ionic gels.

In this integrated configuration, graphene interacts with the evanescent tail of a mode propagating in a dielectric waveguide. This approach enables electrical gating and contacting, allowing for high-speed (> GHz) control over graphene’s chemical potential which support the development of integrated electro-absorption modulators and photodetectors^24,25^. Additionally, this integration method enables scalable and reproducible manufacturing of these devices, as we demonstrated in imec’s 300 mm fabrication facility^26^.

Saturable absorption of graphene sheets placed on dielectric waveguides has been demonstrated on various integrated photonic platforms^27–31^ which have shown the capability to passively mode-lock fiber-lasers^32–34^. Our approach incorporates wafer-scale manufacturing and integrates a gate for electro-optic control of graphene, enabling electrical tuning of graphene’s chemical potential. This introduces an additional parameter for passive mode-locking, providing control over insertion loss and saturable absorption depth^35,36^.

In addition to passive mode-locking, active mode-locking is achieved by modulating the optical cavity loss (or gain) at an integer multiple of the cavity round-trip frequency. To date, actively mode-locked lasers using 2D materials have only been demonstrated in a system where graphene was integrated on a gold reflector with an HfOx gate dielectric, achieving second-harmonic mode-locking at 8.7 MHz^37^. However, applications such as high-speed analog-to-digital converters (ADCs), where actively mode-locked lasers serve as low-jitter clock sources, require repetition rates in the GHz range^38,39^. While transition metal dichalcogenide (TMDC)-based electro-optic modulators have been integrated into photonic circuits at C-band, their low carrier mobility (200 $\text {cm}^{2}\text {V}^{-1}\text {s}^{-1}$)^40^ limits their modulation speeds to below 1 GHz^41^. In contrast, graphene absorbers have demonstrated electro-optic modulation speeds exceeding 10 GHz^24,26^, making them a strong candidate for high-speed mode-locking applications.

In this work, we integrate a single-layer graphene absorber with a commercial erbium-doped fiber amplifier (EDFA) to demonstrate both passive and active mode-locking. Passive mode-locking is achieved at a fundamental repetition rate of 27.9 MHz, producing pulses as short as 1.7 ps. Furthermore, we demonstrate electrical tuning of the absorber’s saturation behavior, allowing compensation for fabrication-induced parasitic doping effects. Active mode-locking is achieved at repetition rates up to 10 GHz, fulfilling the requirements of high-speed ADC systems. The absorber is fabricated on a silicon photonics platform using a wafer-scale CMOS-compatible process, demonstrating a scalable pathway for integrated mode-locking solutions.

## Graphene absorber design

**Fig. 1:**
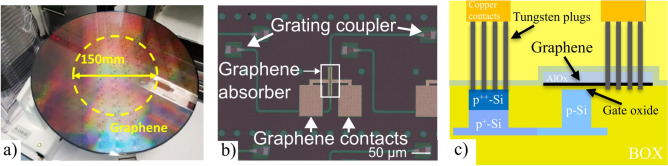
Schematics of the graphene absorber used in this paper, taken from^26^. (**a**) shows a photograph of the wafer during manufacturing, (**b**) shows a top-down microscope image of the device, and c) shows a schematic cross-section of the graphene absorber.

The graphene absorbers used in the experiments described below were manufactured on a wafer scale in imec’s 300 mm fab, utilizing imec’s silicon photonics platform consisting of a 220 nm thick silicon layer on a 2 $\mu$m buried oxide (BOX). Figure 1c schematically shows the cross-section of the single-layer graphene absorber. The optical mode is confined in a 500 nm wide socket waveguide, where a doped silicon waveguide serves as a gate for controlling graphene’s chemical potential. Increasing the doping in waveguides reduces electrical resistance but also induces optical losses. To achieve a balance between lower gate resistance and minimized optical losses three separate ion doping steps were employed^26^.

Graphene, grown via chemical vapor deposition (CVD) on a 6-inch wafer, was transferred to the patterned 300 mm silicon wafer by Graphenea as depicted in Fig. 1a. Raman Spectroscopy on the transferred graphene showed a doping level between $6 \times \ 10^{12} \text { cm}^{-2}$ and $10 \times \ 10^{12} \text { cm}^{-2}$, with a FWHM of the 2D-peak having median values between 35 and 40 $\text { cm}^{-1}$^26^. The position of the 2D peak was found to be between 2675 and 2690 $\text { cm}^{-1}$, which is consistent with the signature of monolayer graphene. A protective layer of 30 nm ALD-AlOx was grown to encapsulate the graphene-AlOx stack which, along with the graphene, was patterned through dry etching using a SiOx hardmask. To enable electrical contacting of the doped silicon and graphene, a pre-metal dielectric was deposited after which via’s were etched. A stack of Ti (titanium), TiN (titanium nitride), and W (tungsten) were deposited after, followed by tungsten polishing and Cu-oxidation steps to form the final metal layer.

Figure1b shows a microscope picture of the top of the single layer graphene (SLG) device. Coupling to and from the SLG absorber was achieved using TE-optimized grating couplers connected through 800 $\mu$m long, 450 nm wide silicon strip waveguides.

A manufactured 100 µm graphene absorber has an electro-optic bandwidth of 11.2 ± 0.7 GHz and an extinction ratio of 5 ± 0.2 dB. More details on the manufacturing and modulation performance can be found in reference^26^.

## Passive mode-locking

Graphene can passively mode-lock lasers through a nonlinear optical phenomenon called saturable absorption, induced by Pauli-blocking^42^. Graphene absorbs light through interband and intraband electronic transitions, which are dependent on the scattering rate, temperature, angular frequency, and Fermi level (or chemical potential) of graphene, and can be calculated using the Kubo equations^43^. In graphene electro-optic modulators the Fermi level is controlled through electro-static gating, which enables electrical tuning of the optical absorption of graphene.

For saturable absorption, absorption coming from optical interband transitions is of primary interest at C-band^15,44–46^. When high-intensity light is incident on the graphene, all possible interband electronic transitions will eventually be blocked, thus reducing graphene’s ability to absorb light. This phenomenon is referred to as Pauli-blocking.

The waveguides used below the graphene are made of silicon, which lacks a direct bandgap and is therefore transparent at C-band, rendering it ineffective as a saturable absorber. However, when silicon is exposed to high optical intensities, two photons can combine via a virtual state to excite a free carrier which induces optical absorption^47^.

In the case of the graphene absorber discussed, these two effects counteract each other: as the intensity increases, the graphene absorption saturates and decreases, while the silicon absorption increases.

Thus, for passively mode-locked lasers, it is uncertain whether graphene saturates sufficiently to achieve the net saturation required for passive mode-locking. Measurements on the net saturation, showing both the graphene saturable absorption and silicon two-photon absorption effects, are discussed in section 3.1. Following this, the capability of a graphene absorber to passively mode-lock a fiber laser is evaluated in section 3.2.

### Saturable absorption

**Fig. 2:**
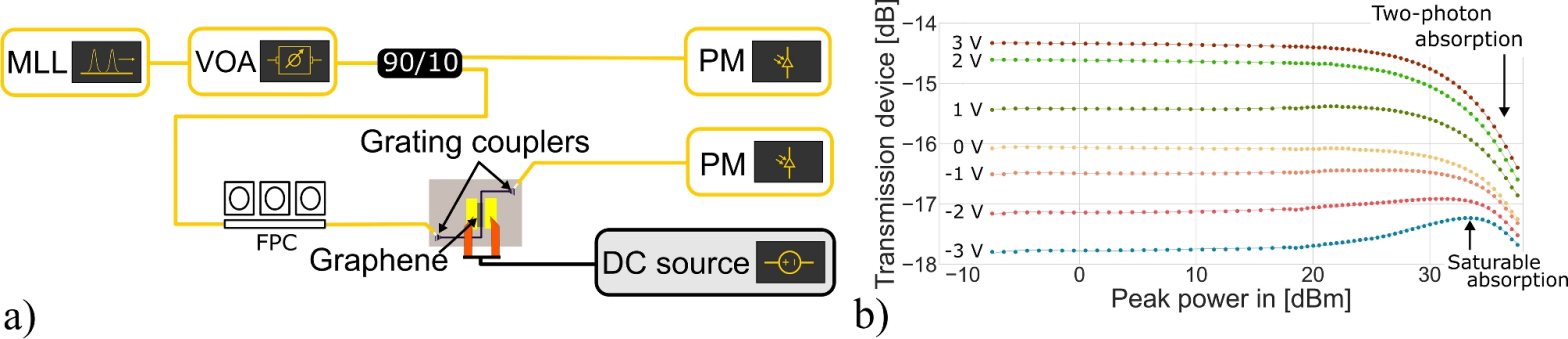
Saturable absorption measurements performed on the single-layer graphene absorber. (**a**) Shows the saturable absorption measurement setup, and (**b**) shows the measured saturable absorption of a 75 µm single-layer graphene absorber, including both grating couplers.

The saturable absorption of the graphene absorber was measured using the measurement setup schematically shown in Fig. 2a. A mode-locked laser (MLL) generating 5 ps sech^2^ pulses with a 2 nm wide optical spectrum centred at 1550 nm was attenuated using a variable optical attenuator (VOA). To measure the transmission of the device, 90% of the attenuated light was coupled through the graphene absorber, and 10 % was used as a reference which are both measured using two Agilent 81532A optical power meters (PM). The graphene absorber was biased by a Keithley source measurement unit.

The transmission of a 75 µm-long graphene absorber was measured for different input powers and bias voltages. The results are presented in Fig. 2b. The peak power on the x-axis corresponds to the pulse peak power before coupling to the chip. The transmission of the device includes the graphene absorber and two grating couplers, each contributing approximately 5.5 dB of loss. The applied bias was limited to a range of -3 V to 3 V to prevent breakdown of the dielectric gate material separating the graphene from the doped silicon waveguide.

The measurements indicate a net saturable absorption effect when the graphene absorber is biased at -2 V or -3 V, within an input peak power range of 15 dBm to 34 dBm. Specifically, when biased at − 2 V, a modulation depth of 1.3 % was observed, with a saturation power of 67.5 mW. For a bias of -3 V, the modulation depth increased to 2.7 %, with a saturation power of 287 mW. Additional details on these calculations are provided in Supplementary Information A. In contrast, little to no saturable absorption was observed for bias voltages between − 1 V and 3 V. This can be attributed to graphene exhibiting the strongest saturable absorption near its neutrality point, which is shifted to negative values due to biaxial strain and doping introduced during manufacturing^26,42^.

Along with the desired saturable absorption that reduces loss at high intensities, Fig. 2b also shows the undesired effect of two-photon absorption caused by the silicon waveguides, in which loss increases with higher optical intensities^47^. This effect becomes more noticeable when graphene is operating in the transparency regime, owing to the increased light intensity in the silicon waveguide after the graphene absorber.

### Passively mode-locked laser cavity

**Fig. 3:**
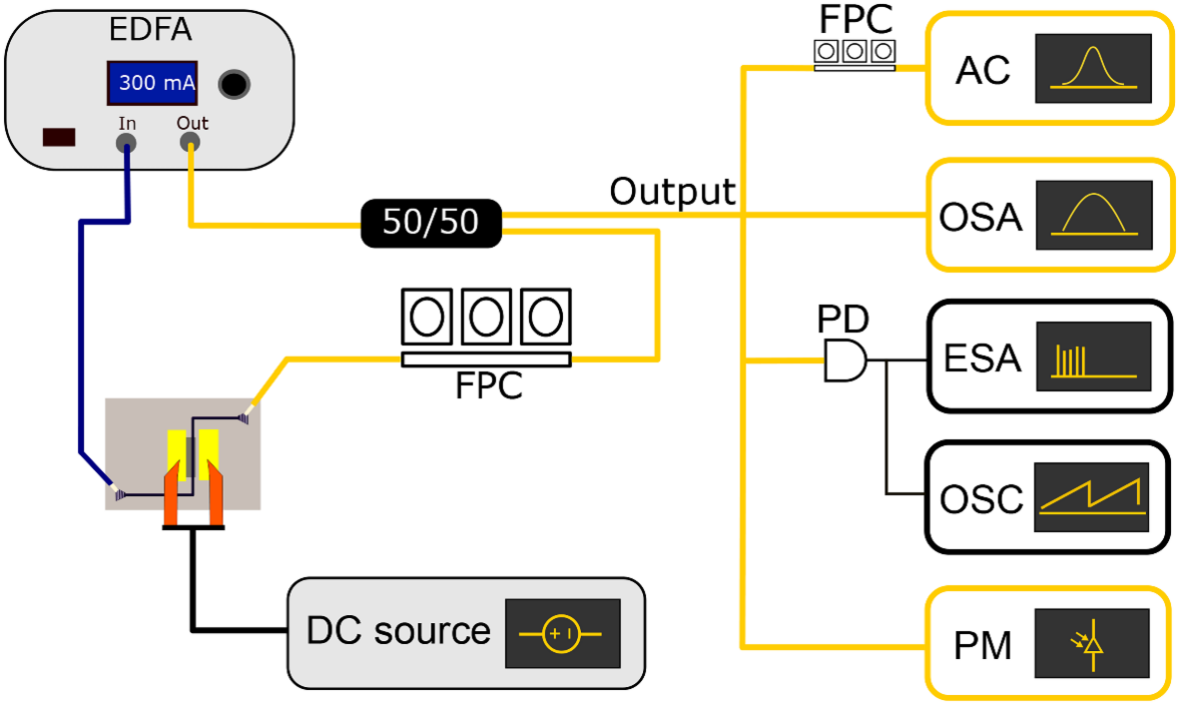
Schematic of the passively mode-locked laser cavity. Yellow fibers represent non-polarization-maintaining (PM) fibers, and blue fibers represent PM fibers.

The graphene absorber was placed inside the laser cavity schematically shown in Fig. 3. This cavity consists of a commercially available Pritel HPP-PMFA-18 erbium-doped fiber amplifier (EDFA), of which the output is passed to a 50:50 out-coupler. A fiber polarization controller (FPC) is used to control the polarization of the light before being coupled to the TE-optimised grating couplers of the graphene absorber. The light is captured from the graphene absorber through a cleaved polarization maintaining (PM) fiber where the polarization maintaining rods are aligned to the TE illumination of the grating couplers. The total cavity length of the mode-locked laser was estimated to be 7 m. Graphene was biased using a Keithley source-measurement unit.

The optical pulse width of the laser was measured using an APE autocorrelator (AC) preceded by a polarization controller having 3 m of fiber. The optical spectrum was measured using a Yokogawa AQ6370 optical spectrum analyser (OSA). The RF spectrum was measured using a Discovery DSCR409 photodetector (PD) connected to a Keysight N9070A electrical spectrum analyser (ESA) and an oscilloscope (OSC), the phase noise was measured using an Advantest ESA. The optical power was measured using an Agilent 81532A power meter (PM).

The procedure used to find stable mode-locking points involved pumping the EDFA and applying a bias to the graphene absorber. The grating couplers on the chip are polarization-dependent; thus, by tuning the polarization controller, the loss in the cavity was adjusted to find a stable fundamental mode-locking position. For the results described in this section, only fundamental mode-locking results are considered. However depending on the configuration, stable higher harmonic mode-locking configurations were also observed.

**Fig. 4:**
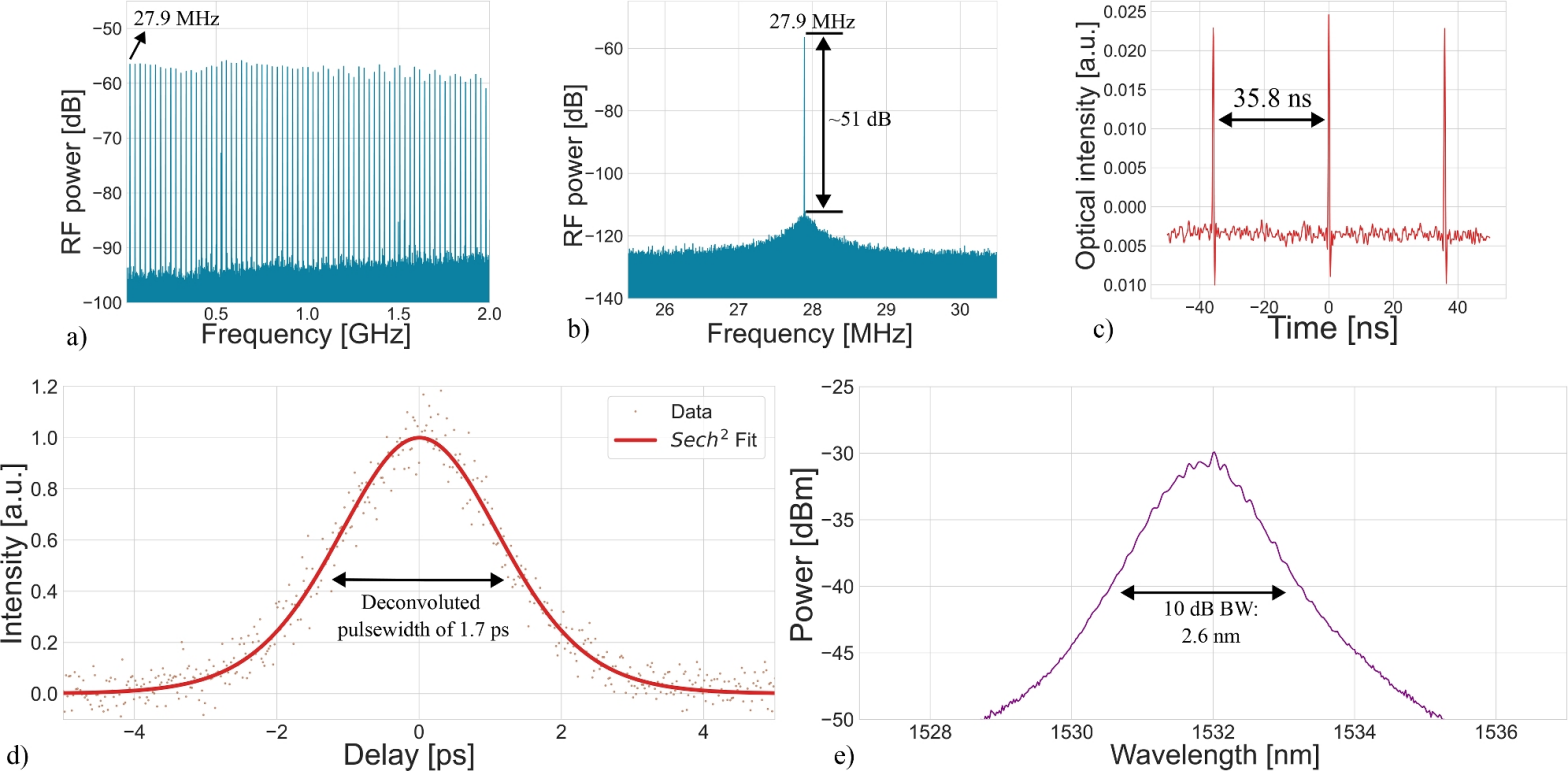
Passive mode-locked laser results with a 75 µm long graphene absorber. The erbium-doped fiber amplifier (EDFA) was pumped at 300 mA, and the saturable absorber (SA) was biased at − 0.7 V. The average optical output power in this configuration is 3 dBm. (**a**) RF spectrum of the generated pulse train. (**b**) Fundamental RF line measured with a 10 Hz resolution bandwidth over a 5 MHz span. (**c**) Pulse train measured using an oscilloscope. (**d**) Autocorrelation measurement fitted with a sech^2^ pulse, showing a deconvoluted FWHM of 1.7 ps. (**e**) Optical spectrum of the passively mode-locked laser with a 2.6 nm optical bandwidth.

Figure 4 shows the measured mode-locking performance for a fundamental mode-locking operating point. Figure 4a,b show a flat frequency comb and 51 dB signal-to-noise ratio of the fundamental RF tone and the pulse train measured using an oscilloscope in Fig. 4c showing pulses having a 35.8 ns spacing indicating stable passive mode-locking at the cavity’s fundamental frequency of 27.9 MHz. In this configuration, the passively mode-locked laser has a deconvoluted pulse width of 1.7 ps as shown in Fig. 4d, a 10 dB optical bandwidth of 2.6 nm as shown in Fig. 4e, and an optical output power of 3 dBm.

**Fig. 5:**
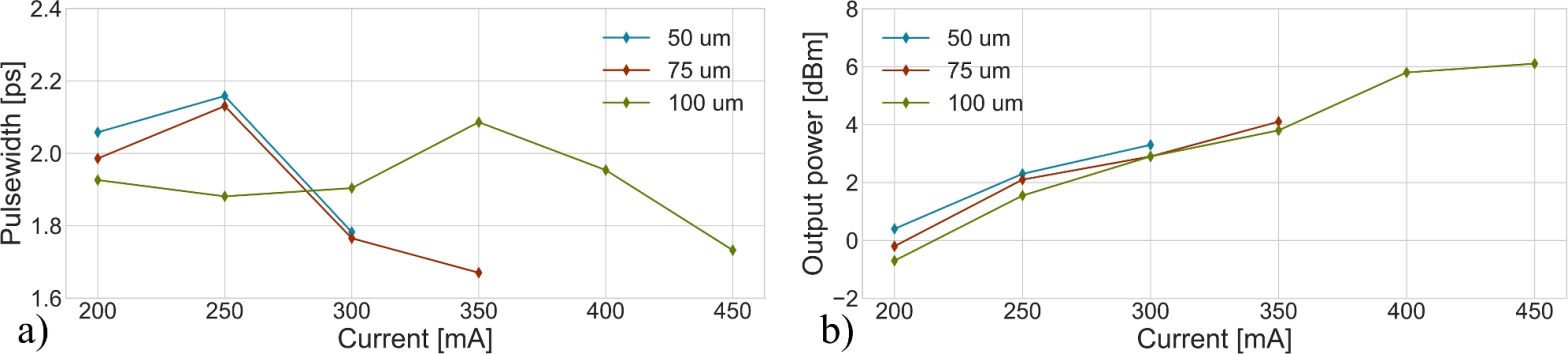
(**a**) Showing the influence of the Pritel pump current and graphene absorber length on deconvoluted pulse-width and (**b**) the average optical output power of the passively mode-locked laser. The graphene absorber bias was fixed at -0.7 V during these measurements.

The influence of the EDFA pump current and absorber length on mode-locking performance is shown in Fig. 5 where the graphene bias was fixed at − 0.7 V. The optical spectrum was not found to change significantly when these parameters were varied, and can be found in the Supplementary Information B. The figure shows the operating combination of absorber lengths and pump currents where fundamental passive mode-locking was possible. Figure 5a shows the influence of these parameters on the pulse width which was found to vary by less than 0.5 ps. The output power was found to increase with the pump current of the EDFA as shown in Fig. 5b. For the 50 µm device no passive mode-locking was achieved beyond 300 mA of pump current. However, the 100 µm device allowed for mode-locking up to a pump current of 450 mA in the EDFA.

An explanation comes from the graphene absorber having a limited range where saturable absorption is present before 2 photon absorption starts to dominate, which is shown in section “Saturable absorption”. Thus, the optical pulse in the cavity should have sufficient power to saturate the graphene device while remaining below the power levels where two-photon absorption losses start to dominate over the saturable absorption effect of graphene. Regarding pulse width, we expect that the main influencing factors are the uncontrolled and unknown dispersion within the laser cavity due to limited knowledge about the fiber inside the EDFA along with spectral shaping effects from the wavelength dependent EDFA gain profile and grating coupler loss. We expect that further dispersion engineering within the fiber cavity can enhance laser performance, as demonstrated previously in the work by Popa et al.^48^.

**Fig. 6:**
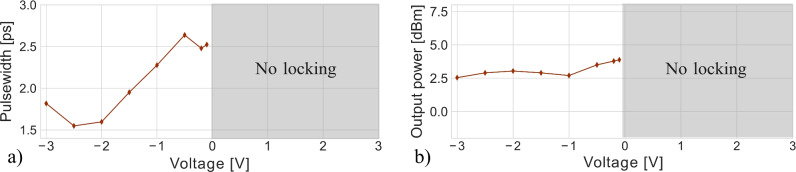
Influence of the applied bias on the graphene absorber with (**a**) showing the deconvoluted pulsewidth and (**b**) output power of the mode-locked laser. The EDFA pump current was fixed at 300 mA, and the graphene absorber length was 75 µm.

The SA measurements discussed in Section “Saturable absorption” show that the saturable absorption depth and absorption of graphene are dependent on the electrical bias applied to the absorber. The influence of the graphene absorber bias on passive mode-locking performance is shown in Fig. 6. Also here, the optical spectrum did not change significantly which is why it was excluded from the figure. These results show that mode-locking is achieved below a voltage of 0 V, which is consistent with saturable absorption only being measured at voltages below 0 V as found in Section “Saturable absorption”. Furthermore, the pulse width was found to increase with higher graphene device bias. This effect could be attributed to a decrease in graphene saturation depth, as discussed in Section “Saturable absorption”.

It should be noted that the pulse width differs by 0.5 ps between Figs. 5a and 6a, despite identical biasing conditions. This variation can be attributed to hysteresis effects caused by defect trapping in the SiOx dielectric spacer between the graphene and silicon waveguide^49^.

## Active mode-locking

The discussed graphene absorbers exhibit electro-optical modulation up to speeds of approximately 11 GHz^26^. In comparison to III/V materials, graphene exhibits notable potential for active mode-locking due to its inherent broadband operation spanning from the visible to the mid-infrared (MIR)^50,51^. Additionally, hybrid mode-locking effects could be achieved when the peak power of the actively mode-locked laser is sufficiently high to saturate the graphene. Until now, active mode-locking using graphene has been limited to the second harmonic frequency (8.7 MHz) of a laser cavity, which was limited by the 13.7 MHz 3 dB electro-optic modulation bandwidth of the graphene modulator used^37^.

This section presents active mode-locking results at higher harmonics (up to 10 GHz), enabled by the high (>10 GHz) 3 dB electro-optic modulation bandwidth of the absorber^26^. In the experiments discussed below, the full potential of graphene’s broad optical bandwidth was not fully utilized, primarily due to the limited optical bandwidth of the grating couplers and the non-uniform gain spectrum of the EDFA used, as shown in Supplementary Information C. This limitation could be addressed in the future by adopting edge-couplers and using gain-flattening filters, which would allow for a wider spectral range and improved performance.

### Nonlinear modulation efficiency

**Fig. 7:**
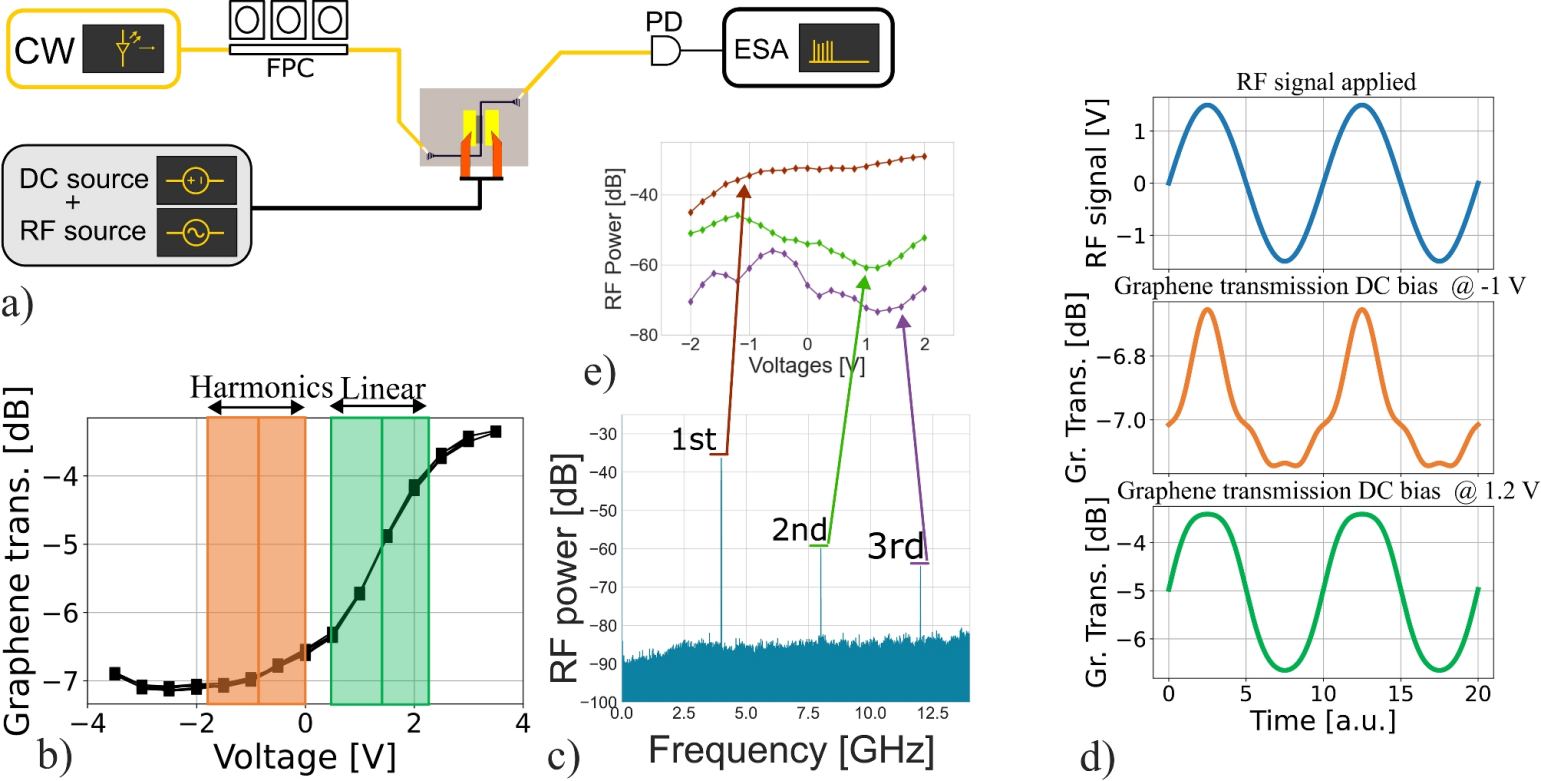
Measurements of distortions generated when driving the graphene absorber with 8.2 dBm RF input at various DC bias values. (**a**) Measurement setup for harmonic generation. (**b**) DC transmission of a 75 µm graphene absorber. (**c**) Electrical harmonics measured using the ESA. (**d**) Simulation showing how a sinusoidal electrical signal (blue) translates into optical distortions at DC biases of 1.2 V (green) and − 1 V (orange). (**e**) Influence of DC bias on the intensity of the 1st, 2nd, and 3rd harmonics.

When using a graphene absorber as an optical modulator for optical data transfer, the device is biased to operate in a linear modulation regime as indicated by the green box in Fig. 7b. In this regime, the electrically applied sinusoidal signal is converted to the optical domain with minimal distortion^26^. However, when the absorber is biased in a nonlinear region or is driven at higher RF powers, as indicated by the orange box in Fig. 7b, distortions occur during the conversion from the electrical to the optical signal which appear in the electrical spectrum as additional harmonics. These translations are schematically depicted in Fig. 7d. The green curve shows the graphene absorption when an RF signal (blue) is applied to the graphene transfer function with a 1.2 V offset (linear modulation regime), while the orange curve represents the graphene absorption with a − 1 V offset (nonlinear modulation regime).

The generated harmonics due to distortions can be measured in the electrical domain using an electrical spectrum analyzer (ESA). The measurement setup, schematically shown in Fig. 7a, was used to measure these distortions. In this setup, a continuous wave (CW) laser is modulated by the graphene absorber at various DC bias voltages and an 8.2 dBm RF signal at a frequency of 4 GHz, the modulated signal is then measured using a Discovery DSCR409 photodiode and a Keysight N9070A ESA. A measured electrical spectrum is shown in Fig. 7c, and the dependence of the generated harmonics on the applied voltage is presented in Fig. 7e. This figure shows that the highest harmonics, and therefore the greatest distortion in the signal translation, occur at a graphene bias of − 1 V.

### Active mode-locking

**Fig. 8:**
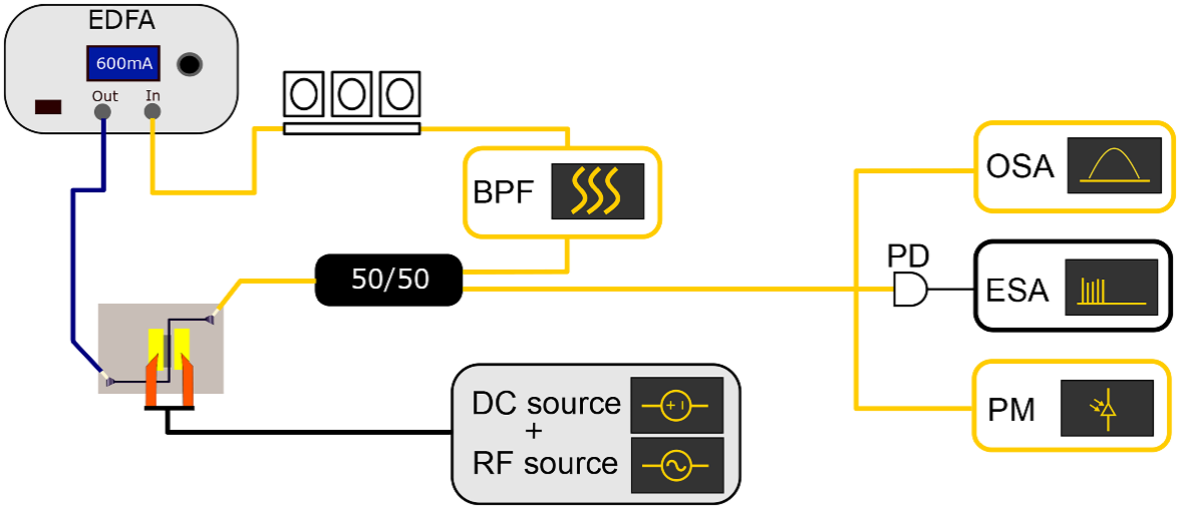
Schematic of the actively mode-locked laser setup.

The actively mode-locked laser cavity is schematically shown in Fig. 8. The cavity consists of a Pritel HPP-PMFA-22 EDFA, pumped at 1000 mA, which guides light to the graphene absorber through a PM fiber aligned to illuminate TE-polarised light on the grating coupler. The output from the chip is coupled to a 50/50 coupler, where half of the light is extracted from the cavity, and the other half is coupled to a Santec OTF-320 24S2 bandpass filter (BPF) with a 1.95 nm optical bandwidth and 10 dB insertion loss, after which the loop is completed with a polarization controller. The total cavity length of the actively mode-locked laser was estimated to be 11 m.

A Yokogawa AQ6370 OSA was used to measure the optical spectrum of the mode-locked laser. A Discovery DSCR409 PD was connected to a Keysight N9070A ESA used to measure the RF spectrum and phase noise of the active mode-locking performance. An Agilent 81532A power meter was used to measure the optical power. The graphene absorber was biased using a Keithley source-measurement unit, and was driven by a R&S SMR40 RF source.

The laser was mode-locked by setting the EDFA pump current, graphene bias and polarization controller. Afterward, the graphene absorber was modulated using the RF signal generator which was fine-tuned to suppress the RF noise floor.

**Fig. 9:**
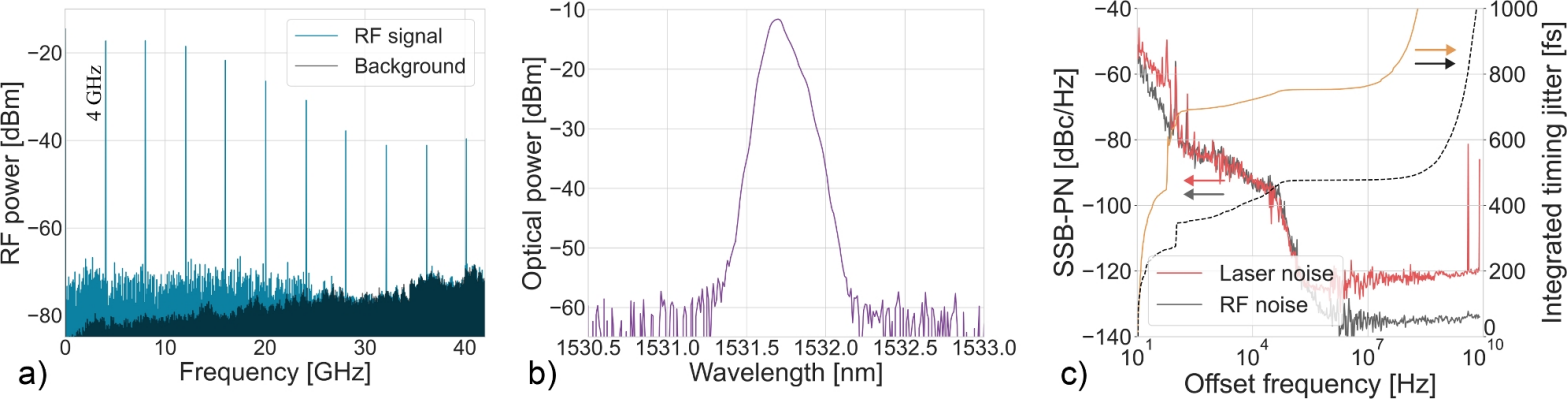
Active mode-locking characterization at 4 GHz. (**a**) RF spectrum, (**b**) single-sideband phase noise, and (**c**) optical spectrum. The absorber is driven with a 15 dBm RF signal and a DC offset of − 1.0 V. The EDFA is pumped at 1000 mA, and the 100 µm graphene absorber produces an optical output power of 1.0 dBm.

**Fig. 10:**
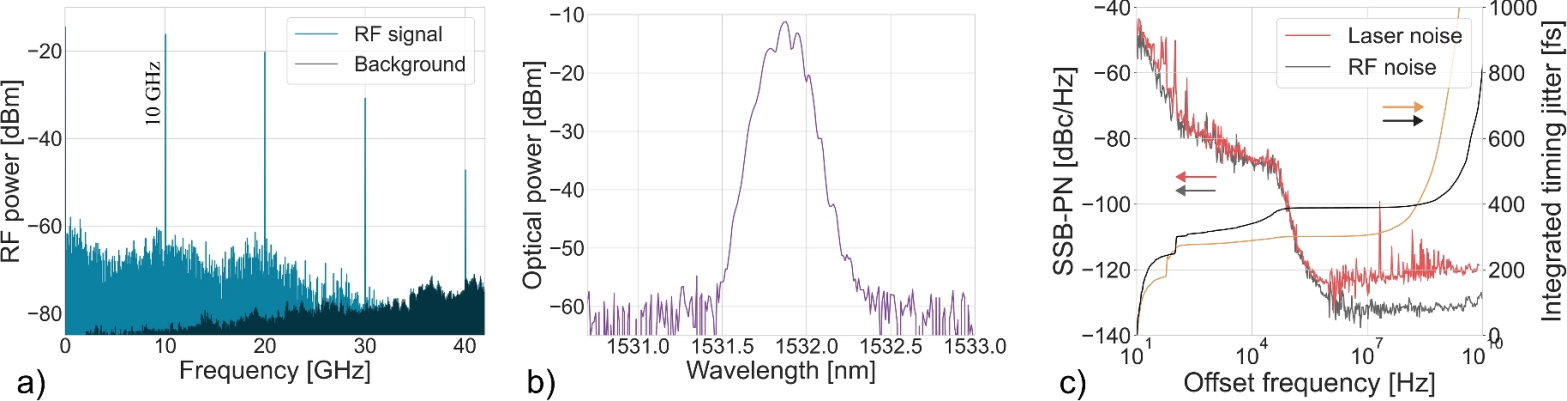
Active mode-locking characterization at 10 GHz. (**a**) RF spectrum, (**b**) single-sideband phase noise, and (**c**) optical spectrum. The absorber is driven with a 13 dBm RF signal and a DC offset of − 0.6 V. The EDFA is pumped at 1000 mA, and the 100 µm graphene absorber produces an optical output power of 1.3 dBm.

The actively mode-locked laser cavity has a fundamental repetition rate of 18 MHz. Through active modulation with an RF signal at 4 and 10 GHz, the mode-locked laser was mode-locked at these respective frequencies, for which the mode-locking spectra are shown in Figs. 9 and 10.The mode locked laser had an average output power of 1.0 dBm in case of the 4 GHz mode-locking and 1.3 dBm in case of 10 GHz mode-locking. The RF spectrum from the laser output is shown in Figs. 9a and 10a showing a 60 dB signal-to-noise ratio on the fundamental RF line. Figs. 9b and 10b show the single-sideband phase noise of the fundamental RF peak. The timing jitter indicated in Fig. 9b and 10b was calculated by integrating the phase noise up the offset frequency, which shows that below 10 MHz the phase noise of the RF source is replicated. The optical spectra are shown in Figs. 9c and 10c. For the active mode-locking results, the spectral width is limited by the BPF used inside the laser cavity, which is necessary for stabilising the mode-locked laser. The optical pulse width could not be measured with the autocorrelator, likely because the peak power of the generated pulse train was too low for detection.

## Discussion and conclusion

The results demonstrate that a graphene absorber integrated on a 220 nm SOI platform is capable of both actively and passively mode-locking fiber lasers, with the absorber being fabricated on a wafer scale. Passive mode-locking produced 1.7 ps pulses at a fundamental repetition rate of 28 MHz. Additionally, active mode-locking was successfully achieved at high harmonics of the fundamental cavity repetition rate, specifically at 4 GHz and 10 GHz.

However, the full optical bandwidth of the graphene saturable absorber could not be fully utilized due to the bandwidth limitations of the grating couplers used, which restricted the 3 dB bandwidth, and the non-flat gain spectrum of the EDFA. This limitation impacted the achievable pulse width of the mode-locked laser. To overcome these constraints, transitioning to edge-coupled integrated graphene absorbers and graphene absorbers integrated on silicon nitride waveguides would enable broader optical bandwidths, enhance performance, and potentially allow for higher optical output powers and reduced noise in mode-locked fiber lasers.

Furthermore, integrating these graphene saturable absorbers with ion-implanted^11,12^ or titanium-sapphire^10^ waveguides could provide a pathway toward fully integrated femtosecond laser sources on-chip.

## Supplementary Information

Supplementary Information.

## Data Availability

All data used to generate the figures in this study are available from the corresponding author on reasonable request.
