# Supplementary material for: Graphene absorber on an SOI chip for active and passive mode locking of lasers
Source: Sci Rep. 2025 Mar 19;15:9399. doi: 10.1038/s41598-025-93051-z (PMC11920391; doi:10.1038/s41598-025-93051-z)
Supplement: Supplementary file 1 — Supplementary Information. [file 41598_2025_93051_MOESM1_ESM.pdf]

Supplementary information: Graphene Absorber on an SOI Chip for Active and Passive Mode Locking of Lasers

Tom Reep[1,2*], Cheng-Han Wu[1,2], Didit Yudistira[2], Steven Brems[2], Inge Asselberghs[2], Marianna Pantouvaki[2], Joris Van Campenhout[2], Dries Van Thourhout[1,2], and Bart Kuyken[1,2]

[1]Photonics Research Group, Department of information Technology, Ghent University-imec, Technologiepark-Zwijnaarde 15, 9052 Gent, Belgium
[2]Imec, Kapeldreef 75, 3001 Leuven, Belgium
[*]Tom.Reep@UGent.be

A Saturation intensity calculations

The saturable absorption measurements in Fig. 2b depict the transmission of the entire graphene absorber, including both grating couplers. The x-axis represents the input peak power before accounting for grating coupler losses. These coupling losses, which are not intrinsic to the graphene device, could be significantly reduced to less than 1 dB by transitioning to alternative coupling methods, such as edge couplers. Therefore, it is essential to extract the intrinsic saturable absorption characteristics of the graphene-on-silicon waveguide.

To isolate the intrinsic graphene behavior, the grating coupler losses were normalized out of both the transmission and input power, with a focus on the -3 V and -2 V bias levels, where the majority of saturable absorption occurs. The resulting absorption curves, plotted on a linear scale in Fig. A1, were fitted using the two-level saturation model:

$$-\mathrm{T(P)} = \alpha(P) = \frac{\alpha_s}{1+\frac{P}{P_{\mathrm{sat}}}} + \alpha_{\mathrm{ns}}, \tag{1}$$

where T(P) is the transmission of the device, α_s is the saturable loss, α_{ns} is the nonsaturable loss, and P_{sat} is the saturation power. The fitting results, excluding the two-photon absorption region, are summarized in Tab. 1 and are plotted as the dashed lines in Fig. A1. The graphene absorber exhibits saturation powers of 287 mW at a -3 V bias and 67.5 mW at a -2 V bias.

The modulation depth of the graphene absorber is limited to 2.7% for a -3 V bias and 1.3% for a -2 V bias, primarily due to two-photon absorption in the c-Si waveguides. Transitioning to SiN waveguides could mitigate this effect, as SiN has significantly lower (negligible at C-band) two-photon absorption effects, thereby enhancing the graphene absorbers modulation depth.

Graph. bias	α_{ns}	α_s	P_{sat}	I_{sat} mode	I_{sat} graphene	Modulation depth
-3 V	6.0 dB	0.86 dB	287.4 mW	1.44 $\frac{PW}{m^2}$	1.12 $\frac{PW}{m}$	2.7 %
-2 V	6.0 dB	0.28 dB	67.5 mW	0.44 $\frac{PW}{m^2}$	0.26 $\frac{PW}{m}$	1.3 %

Table 1. Fit parameters of the 2-level saturation equation shown in eq. A shown as the dashed line in Fig. A1

A.1 Calculating the saturation intensity of the mode

The saturation intensity of the graphene absorber was calculated by first calculating the optical mode using a mode solver. The normalised field magnitude was calculated from the normalised electric $\tilde{\mathbf{E}}$- and magnetic $\tilde{\mathbf{H}}$-fields as

$$\tilde{\mathbf{S}}(x,y) = \frac{1}{2}\mathrm{Re}\left\{\tilde{\mathbf{E}}(x,y)\times\tilde{\mathbf{H}}(x,y)^*\right\} \tag{2}$$

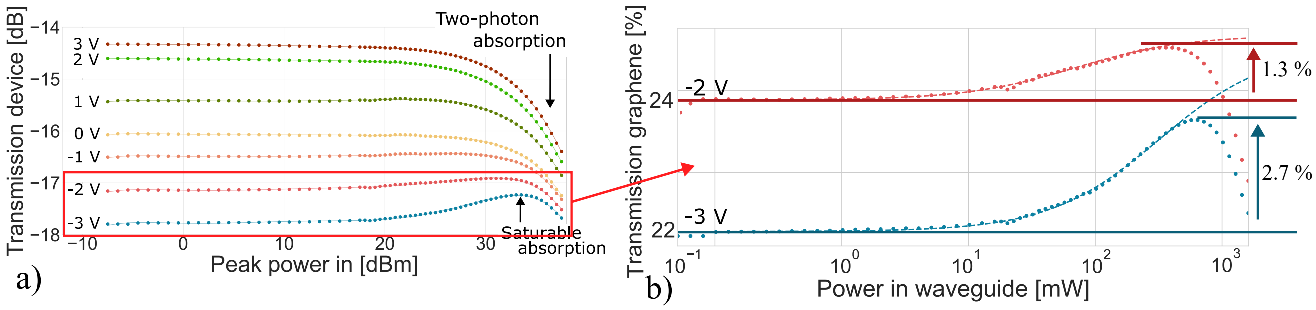

Figure A1. Fig. A1: a) Saturable absorption curve previously shown in Fig. 2 in the manuscript, and b) highlighted saturable absorption curves for -3 V and -2 V biases applied to the graphene absorber. The dashed lines represent the fitted curves using the two-level saturation model (Eq. A).

which represents the normalised power flow per unit area (Poynting vector) of the optical mode. This quantity is unitless since no normalisation has been performed after the mode calculation. This normalised field magnitude is shown in Fig. A2a).

From this normalised field magnitude, the effective area was calculated using the intensity of the electric fields[1], defined as

$$A_{\text{eff}} = \frac{\left(\iint |\tilde{\mathbf{E}}(x,y)|^2 \, dx\, dy\right)^2}{\iint |\tilde{\mathbf{E}}(x,y)|^4 \, dx\, dy} \tag{3}$$

which results in an effective mode area of $0.200\,\mu\text{m}^2$.

The saturation intensity was derived from the fitted saturation power P_{sat}, which is 287.4 mW at -3 V and 67.5 mW at -2 V. This corresponds to optical mode saturation intensities of 1.44 PW/m^2 and 0.44 PW/m^2, respectively. However, these values represent the saturation intensity of the entire mode and do not account for the local intensity at the graphene interface, which depends on the waveguide geometry, material, and the graphene's position above the waveguide.

A.2 Calculating the saturation intensity at graphene interface

The actual intensity of the optical light at the graphene interface is of greater interest. This intensity was calculated by taking a slice of the normalised E-field vector $\tilde{\mathbf{E}}(x,y)$ at the graphene interface (located 5 nm above the c-Si waveguide, corresponding to the gate thickness in our device) and calculating the effective width of this slice. The effective width is defined as

$$w_{\text{eff}} = \frac{\left(\int |\tilde{\mathbf{E}}(x)|^2 \, dx\right)^2}{\int |\tilde{\mathbf{E}}(x)|^4 dx} \tag{4}$$

The calculated effective width of the mode profile slice shown in Fig. A2b), is 406 nm. The saturation intensity at the graphene interface is then calculated as

$$I_{\text{sat}} = \frac{P_{\text{sat}}}{w_{\text{eff}}} \frac{\int \tilde{\mathbf{S}}(x)\, dx}{\iint \tilde{\mathbf{S}}(x,y)\, dx\, dy} \tag{5}$$

where $\tilde{\mathbf{S}}(x)$ is the normalised Poynting vector 5 nm above the c-Si waveguide. This results in graphene saturation intensities of 1.12 PW/m and 0.26 PW/m at -3 V and -2 V, respectively.

B Passively mode-locked laser: optical spectrum

In the paper, section 3.2 discusses the effects of pump current and graphene bias on the performance of the passively mode-locked laser. As mentioned in the text, the optical spectrum was found to remain relatively unchanged across different operating conditions. To further illustrate this, Fig. A3 shows the optical spectra for the passive mode-locking regimes under various pump currents.
The spectra demonstrate that the central wavelength and spectral shape remain largely unaffected by changes in pump current.

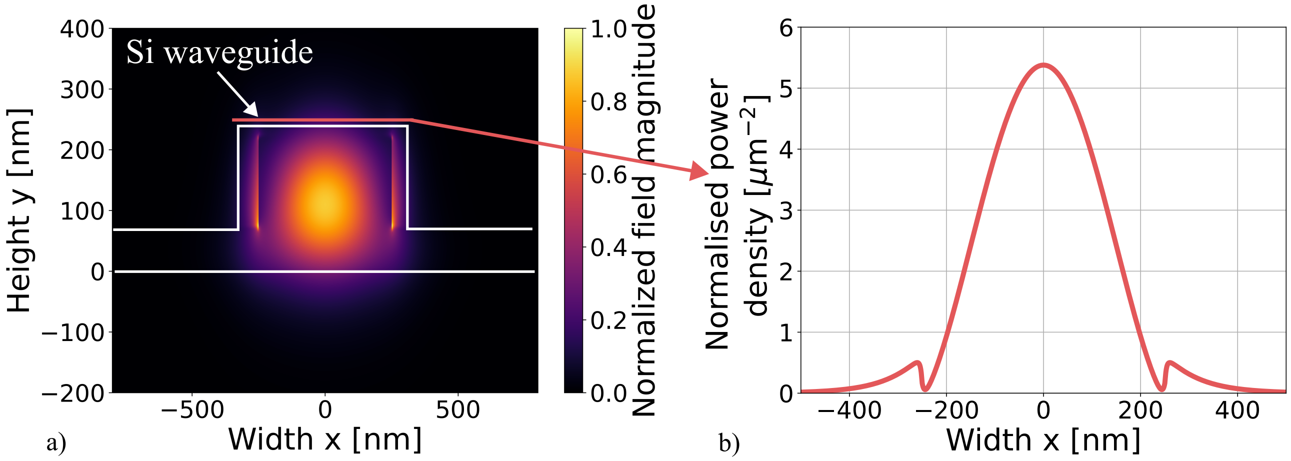

Figure A2. Optical mode of the c-Si waveguide. Fig. a) shows the optical mode used to calculate the effective mode area, being 0.200 μm^2. Fig b) shows a slice from the optical mode at a height of 225 nm (see red line) normalised giving the normalised power density. This was used to calculate the effective graphene width being 406 nm.

C Gain spectrum of the Pritel HPP-PMFA-22-10

The Pritel HPP-PMFA-22-10 EDFA has a non-uniform gain spectra. The gain spectrum of a Pritel HPP-PMFA-22-10 EDFA when pumped with a current of 1000 mA is shown in Fig. A4.

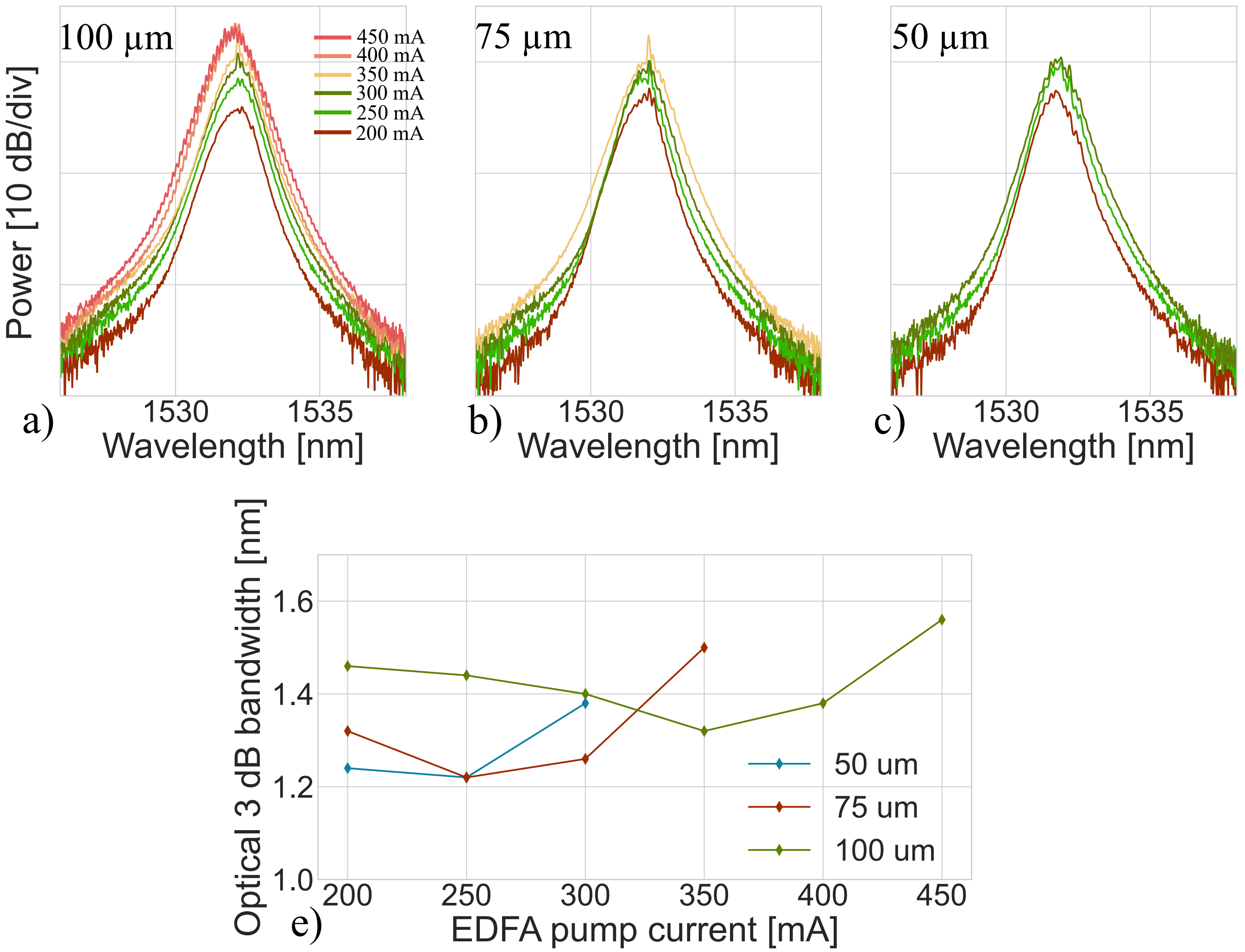

Figure A3. The optical spectra variations depending on the EDFA pump current. Fig. a) shows the passive mode-locking spectra for the 100 μm graphene absorber, b) the spectra for a 75 μm absorber and c) for a 50 μm absorber. Fig. d) shows the optical 3 dB bandwidth plotted against pump current.

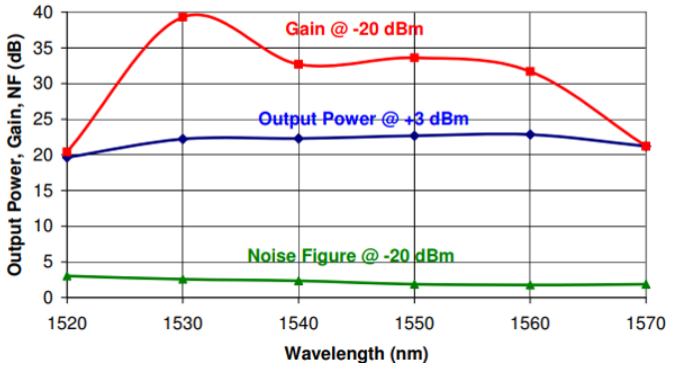

Figure A4. The gain spectra of the Pritel HPP-PMFA-22-10 EDFA when pumped with a pumping current of 1000 mA.

References

1. Agrawal, G. Chapter 2 - pulse propagation in fibers. In Agrawal, G. (ed.) *Nonlinear Fiber Optics (Fifth Edition)*, Optics and Photonics, 27–56, DOI: https://doi.org/10.1016/B978-0-12-397023-7.00002-4 (Academic Press, Boston, 2013), fifth

edition edn.
